# Multi-Mycotoxin Analysis in Durum Wheat Pasta by Liquid Chromatography Coupled to Quadrupole Orbitrap Mass Spectrometry

**DOI:** 10.3390/toxins9020059

**Published:** 2017-02-09

**Authors:** Josefa Tolosa, Giulia Graziani, Anna Gaspari, Donato Chianese, Emilia Ferrer, Jordi Mañes, Alberto Ritieni

**Affiliations:** 1 Laboratory of Food Chemistry and Toxicology, Faculty of Pharmacy, University of Valencia, Av. Vicent Andrés Estellés s/n, Burjassot, València 46100, Spain; emilia.ferrer@uv.es (E.F.); jordi.manes@uv.es (J.M.); 2 Department of Pharmacy, Faculty of Pharmacy, University of Naples “Federico II”, Via Domenico Montesano 49, 80131 Napoli, Italy; giulia.graziani@unina.it (G.G.); annagaspari@virgilio.it (A.G.); donato.chianese@gmail.com (D.C.); alberto.ritieni@unina.it (A.R.)

**Keywords:** baby-food, durum wheat pasta, multi-residual method, HRMS-orbitrap

## Abstract

A simple and rapid multi-mycotoxin method for the determination of 17 mycotoxins simultaneously is described in the present survey on durum and soft wheat pasta samples. Mycotoxins included in the study were those mainly reported in cereal samples: ochratoxin-A (OTA), aflatoxin B_1_ (AFB_1_), zearalenone (ZON), deoxynivalenol (DON), 3-and 15-acetyl-deoxynivalenol (3-AcDON and 15-AcDON), nivalenol (NIV), neosolaniol (NEO), fusarenon-X, (FUS-X), T-2 toxin (T-2) and HT-2 toxin (HT-2), fumonisin B_1_ and B_2_ (FB_1_ and FB_2_), and four emerging mycotoxins: three enniatins (ENA, ENA_1_, and ENB), and beauvericin (BEA). Twenty-nine samples were analyzed to provide an overview on mycotoxin presence: 27 samples of durum wheat pasta, and two samples of baby food. Analytical results concluded that trichothecenes showed the highest incidence, mainly DON, NIV, and HT-2 toxin, followed by ZON and ENB, while NEO, FUS-X, OTA, AFB_1_, and FUM were not detected in any sample. The highest contents corresponded to ENB and ranged from 91.15 µg/kg to 710.90 µg/kg.

## 1. Introduction

Cereal-based products represent one of the most important dietary items in many countries around the world, mainly those from wheat, which is the most consumed cereal worldwide [1]. Usually, wheat is ground to flour for the production of bread, pasta, biscuits, and other products. Durum wheat (*Triticum durum Desf*.) is the most widespread crop in the Mediterranean area. Thus, sixty-seven per cent of the Italian durum wheat production, mainly from the southern regions, is used for producing pasta [2].

Unfortunately, infection of crops and stored cereals with fungi can result in the production of secondary toxic metabolites known as mycotoxins. These hazardous natural toxins can be transferred into the final products, which constitute a main concern for all steps involved in food safety issues due to its implication on human and animal health [3,4]. Furthermore, available data indicated that durum wheat was generally more contaminated than common wheat [5].

Mycotoxins are toxic secondary metabolites produced by filamentous fungi, mainly *Aspergillus* spp., *Penicillium* spp. and *Fusarium* spp. [6]. Currently, diverse mycotoxins have been identified, but the most important ones regarding their occurrence and toxicity are aflatoxins (AFs), fumonisins (FMs), trichothecenes (TRC), ochratoxins (OTs), patulin (PAT), and zearalenone (ZON) [4].

ZON and its metabolites are estrogenic mycotoxins commonly found in maize, but barley, oats, wheat, rice, sorghum, and soy beans are also susceptible of contamination with *Fusarium* (*F. graminearum*). Those raw cereals, are often contaminated with type B-TRC particularly deoxynivalenol (DON) and nivalenol (NIV). DON is the most prevalent TRC worldwide. TRC are a family of related sesquiterpenoids and according to their functional groups are divided into four groups (A–D). HT-2, T-2 toxins, diacetoxyscirpenol (DAS) and neosolaniol (NEO) are type A TRC. Type B TRC are represented by DON and its derivates 3-acetyl-deoxynivalenol (3-AcDON) and 15-acetyl-deoxynivalenol (15-AcDON) and also fusarenon-X (FUS-X) and NIV. The most common TRC found in cereal and cereal products include: NIV, DON, FUS-X, 15-AcDON, 3-AcDON, DAS, NEO, HT-2, and T-2 toxins [7]. In this sense, *Fusarium* mycotoxins have been reported as the most prevalent in cereal foodstuffs, mainly type B TRC. 

Legislation on the maximum permitted limits in many raw and processed products, mainly for cereals and cereal-based products, has been established by the Commission Regulation (EC) No. 1881/2006 [8] and its amendments. These limits have been set for DON, the key representative of group B TRC, the sum of fumonisin B_1_ (FB_1_) and fumonisin B_2_ (FB_2_), ZON, AFs, ochratoxin A (OTA), and PAT. For two other *Fusarium* mycotoxins, HT-2 and T-2 toxin, indicative levels have been recommended in cereals and cereal based products [9].

In addition to those regulated *Fusarium* mycotoxins, other “emerging mycotoxins”, such as enniatins (ENs) and beauvericin (BEA), have been reported in cereals mainly in Northern Europe (Norway and Finland) [10], but also in Central and Southern Europe, especially in the Mediterranean area [11]. ENs and BEA are produced by different *Fusarium* species, such as *Fusarium verticillioides*, *Fusarium proliferatum*, *Fusarium subglutinans*, *Fusarium oxysporum*, *Fusarium poae*, and *Fusarium avenaceum*. These molds are common in areas of Central and Southeastern Europe and can affect many important food commodities [12]. The level of contamination reported in literature varies considerably worldwide according to geographical area, region and year, which ranges from a few ng/g to several µg/g. These mycotoxins have been reported in cereal based products [3] and in pasta samples in higher contents, up to µg/g [13].

Up to now, despite the wide presence of these mycotoxins in different foodstuffs, limited knowledge is available on the transfer of ENs across the food chain and no maximum limits (MLs) are currently available for emerging *Fusarium* mycotoxins. Thus, monitoring studies for them are necessary for legislative purposes, in order to establish an appropriate maximum contamination level by the authorities [14].

With the aim of detecting as many mycotoxins as possible in a single run, multi-mycotoxin methods have been commonly employed in last years [15,16,17,18]. These multi-mycotoxin methods often include the analysis of TRC, FUM, and/or ZON, as well as OTA, AFs, and other toxins. All these mycotoxins show very different chemical and physicochemical properties and behaviors and can be present in various matrix–toxin combinations.

Thus, multi-mycotoxin method development employing the same sample preparation and a single final determination is a challenge. In this sense, the most critical step is the extraction, which should allow good recoveries for all mycotoxins under investigation in a specific food matrix. Therefore, it is necessary to develop multi-toxin methods for the simultaneous analysis of emerging and traditional mycotoxins. To our knowledge, this is the first report on multi-mycotoxin analysis in pasta samples by orbitrap mass spectrometry.

Within this context, the aim of the study was to determine the incidence and levels of common mycotoxins (AFB_1_, OTA, NIV, NEO, FUS-X, FB_1_, FB_2_, DON, 15-AcDON, 3-AcDON, HT-2, T-2, ZON, ENB, ENA, ENA_1_, and BEA) found in pasta samples available on the Italian market, by using one single extraction method and comparing the results with the ML established by the EU. Determination has been carried out by UHPLC-orbitrap mass spectrometry.

## 2. Results

### 2.1. Method Implementation: Optimization of the UHPLC-Q-Orbitrap Conditions

First, in order to evaluate chromatographic conditions, several experiments were performed on different gradients of mobile phases. Better results were obtained when initializing with 60% phase B. The optimum mass spectrometric parameters for the identification and quantitation of the seventeen analytes were first obtained after analyzing the standard work solution at 100 µg/L. Sensitivity was checked by recording full scan chromatograms in both positive and negative ionization modes. Due to adduct formation with formic acid/ammonium formate buffer, some analytes exhibit strong formic or ammonium adduct species ([M + FAc]^−^ or [M + NH_4_]^+^, respectively) which appear to be the most predominant ions in the mass spectrum (Table 1).

### 2.2. Validation Parameters

Recoveries were performed by adding fortification standards to samples at three concentration levels (125 µg/kg, 62.5 µg/kg, and 12.5 µg/kg) using two grain samples (durum wheat, and soft wheat). This procedure was carried out in triplicate. As reported in scientific literature, spiked matrices are not affected by the conditions occurring in naturally-contaminated samples, where the mycotoxins are fixed in the matrix due to the fungi production in the substrate. Thus, the procedure of spiking blank matrices may not simulate the real extraction efficiency, due to the fact that mycotoxins are only applied to the surface of the matrix [19].

For this reason, recovery assays were performed in naturally-contaminated samples, taking into account the mycotoxin amount in samples. In this sense, to the final observed concentration after addition, the mycotoxin amount naturally present in the samples had been subtracted. Then, the recovery quantitation was performed using calibration curves constructed in solvent and then corrected by the matrix effect. The mean recoveries calculated for all mycotoxins and for both matrices at the three fortification levels are provided in Table 2.

The accuracy was estimated by calculating the recovery for each compound. Recovery experiments were conducted at three different levels for each matrix as described in Section 5.7.1. The mean recovery values ranged between 67%–206%, 67%–213%, and 69%–210%, respectively. In Table 2, the recovery values represent the average obtained from each spiked level performed in triplicate (*n* = 9). Intraday precision was determined by calculating the relative standard deviation (RSDr), obtained from results generated under repeatability conditions of six determinations for each spiked sample in the same day. RSDr for the validated procedures at each spiked level were lower than 8%, 6%, and 5%, respectively. The same approach was applied for the determination of the interday precision, differing in three days instead of one day. The interday precision was calculated by the relative standard deviation (RSDr) from spiked samples under reproducibility conditions by one determination per concentration on three consecutive days. RSDr for the validated procedures at each spiked level were lower than 13%, 11%, and 8%, respectively.

### 2.3. Matrix Effect Studies

Matrix effects for mycotoxins analyzed in the study are showed in Table 2. As it can be observed, except for BEA and some TRC, ME were close to 100%. The matrix effect (signal enhancement or suppression) was investigated by calculating the ratio percentage between the slopes of the matrix-matched calibration curve and the curve in solvent. To correct the matrix effects, matrix-matched calibration curves were constructed initially in the matrix for quantification purposes. However, as it was not possible to find a blank sample for all analyzed mycotoxins, calibration standards were dissolved in solvent, in order to avoid undesirable interactions between naturally occurring mycotoxins and spiked ones. For this reason, the final amount was corrected by the calculated matrix effect for each mycotoxin.

### 2.4. LOD, LOQ, and Linearity

Sensitivity was evaluated by limit of detection (LOD) and limit of quantitation (LOQ) values. The LODs and LOQs were determined as described in Section 5.7.3. Calculated LOD and LOQ are shown in Table 2. As can be observed, LOD and LOQ for legislated mycotoxins were lower than the ML established. Furthermore, according to Commission Decision EC/2002/657 [20], all mycotoxins exhibited a good linearity in the working range as it is shown in Table 2 expressed as “*r*^2^”.

### 2.5. Mycotoxin Occurrence

A multianalyte method employing ultra high performance liquid chromatography coupled with orbitrap high-resolution mass spectrometry has been applied for monitoring cereal-based products. Thus, the proposed method has shown to be suitable to investigate the occurrence of seventeen mycotoxins in a total of twenty-nine commercial pasta (durum wheat and soft wheat baby food). To our knowledge, this is the first report on multi-mycotoxin analysis in pasta samples by orbitrap mass spectrometry. 

The mycotoxin incidence and contents in pasta samples, as well as MLs established, are presented in Table 3. As it can be observed, DON was the prevalent toxin (100%), followed by NIV, ZON, ENA_1_ (93%), HT-2, and ENB (90%). In the present survey, DON also showed higher contents, which ranged from 20.89 µg/kg to 247.27 µg/kg. Data reported in the present study about DON contamination levels were all very far from the maximum limit (1750 μg/kg) set for unprocessed durum wheat [8]. For NIV, 93% of pasta samples analyzed were positive with an average amount of 15.33 µg/kg. Referent to 3-AcDON and 15-AcDON, scarce literature has been reported regarding wheat contamination. In our study, 87% of wheat samples were positive for the sum of 3-AcDON and 15-AcDON with a mean value of 2.47 µg/kg. Regarding HT-2 and T-2 toxin, mean contents of 36.95 μg/kg and 12.46 μg/kg for T-2 and HT-2, respectively, were found. 

Although ZON occurrence has been widely described mainly in maize samples, wheat and other cereals can be also contaminated by this estrogenic mycotoxin. In the present study, ZON was detected in 93% of samples and contents ranged from 16.84 µg/kg to 19.94 µg/kg. The levels found did not exceed the ML established (20–75 µg/kg). Regarding emerging *Fusarium* mycotoxins, our results showed that ENs were present in 72% of pasta samples analyzed. The percentages for each EN are reported in Table 3, as well as mean values. As it can be observed, ENB showed the highest incidence (90%) and also the highest content reported for all mycotoxins included in the study (710.90 µg/kg). The mycotoxins OTA, NEO, FUS-X, AFB_1_, and FUM, were not found in the present study, although their presence has been reported by other researchers in cereal samples [21,22].

#### 2.5.1. Mycotoxin Occurrence in Baby Food

Regarding results obtained for baby food samples analyzed, although only two samples were included in the survey, mean contents for some mycotoxins were higher in baby food than in durum wheat pasta samples. In this sense, DON presented a mean value of 103.82 µg/kg and the highest content was 124.55 μg/kg. DON was also one of the mycotoxin with highest incidence together with 3-AcDON, 15-AcDON, ZON and ENA_1_. HT-2 and T-2 toxin were only detected in one out of two samples of baby food analyzed, with 7.60 µg/kg and 27.54 µg/kg, respectively. 3-AcDON and 15-AcDON were present in baby food samples analyzed in our survey, but showed lower contents (2.46 µg/kg), similar to those reported for these mycotoxins in durum wheat samples analyzed in our study (2.12 µg/kg). For ZON, contents reported in baby food were the same than contents in durum wheat pasta, 17.52 µg/kg and 17.54 µg/kg, respectively. However, although NIV was present in durum wheat pasta samples, no sample of baby food analyzed were positive for this TRC.

#### 2.5.2. Mycotoxinco-Occurrence

The simultaneous presence in the same commodity of mycotoxins produced by fungi belonging to different genera is not uncommon [23]. In this sense, given the simultaneous presence of emerging *Fusarium* mycotoxins commonly reported in literature and combinations with the so called traditional *Fusarium* mycotoxins (TRC, FMs, etc.) found by many authors in foodstuffs, special focus should be paid to the simultaneous presence of various mycotoxins in a sample, as synergistic effects have been reported in literature [24].

The co-occurrence of more than two mycotoxins in a single sample has been evidenced, representing a possible health risk than the intake of only one mycotoxin alone due to the combined intake of mycotoxins. In this sense, more than 80% of each sample analyzed were contaminated with 6–10 mycotoxins.

## 3. Discussion

In the last years, increasing *Fusarium* incidence and a higher DON accumulation in durum wheat has been reported in Italy, especially in northern regions. Its occurrence in durum wheat increases from southern to northern areas in Italy, with a heavy influence of some factors, such as year and area of cultivation [2,7].

Results found in our survey (Table 3) are in accordance with those reported by other authors, who found DON as the most prevalent mycotoxin in wheat and wheat-based foodstuffs [22,25,26,27,28,29,30] and pasta samples [31,32]. These results were also reported by Rodríguez-Carrasco et al. [21] in semolina samples; however, these authors reported lower DON contents than those found in our study. Regarding NIV occurrence, the values obtained are similar to those found by other researchers, although they reported lower incidences, but contents were in the same range. In this sense, according to Rodríguez-Carrasco et al. [21], the 20% of semolina samples were positive for NIV with an average amount of 10.9 µg/kg. Malachova et al. [3] reported a mean value of 30 μg/kg for NIV. However, Jestoi et al. [10] and Lattanzio et al. [33] reported higher mean contents in wheat samples, 150 µg/kg, and 63.5 µg/kg, respectively, which were higher than those found in our survey.

Regarding 3-AcDON and 15-AcDON contents, similar results were reported by Jestoi et al. [10] and Rodríguez-Carrasco et al. [21], who found an average of 17 µg/kg in Finnish grain samples, and 4.4 µg/kg in semolina, respectively. On the other hand, higher contents for the sum of 3-AcDON and 15-AcDON were reported by Bryła et al. [30].

For HT-2 toxin, similar contents to those obtained in our study were reported by other authors [21,33,34,35,36]; however, in the study conducted by Cano-Sancho et al. [34], contents up to 46 µg/kg were reported. Regardin ZON contamination, lower incidence was reported by Juan et al. [37], who found ZON in 9% of wheat samples. Levels were well below the ML established. Nevertheless, in the study reported by Bryła et al. [30], level was exceeded in a single sample of grain from Osiny (Poland) which presented 100 µg/kg, although the mean average reported was 43 µg/kg.

Regarding emerging *Fusarium* mycotoxins, ENs and BEA, the occurrence of relatively high levels (up to mg/kg) of these mycotoxins in cereal grains, has been reported in some studies conducted in Europe [10,38]. In this sense, Uhlig et al. [39] reported an incidence of 100% for ENB in grain samples. ENB was also the most abundant mycotoxin found pasta samples analyzed by Serrano et al. [38]. However, in the study reported by Malachova et al. [3], ENA was detected in 97% of samples (concentration range of 20–2532 μg/kg) followed by ENB and ENB_1_ (91% and 80%, respectively), while ENA_1_ was found only in 44% of samples. Similar results were reported by Juan et al. [40] who reported an ENB prevalence of 44% and levels up to 106 µg/kg. Concerning BEA, lower incidence was reported by different authors [38,40].

Special attention should be paid in contents reported in baby food samples because children are considered a vulnerable population group more susceptible to mycotoxin exposure than adults, since they have a restricted diet and they consume more food on a body weight basis than adults. Additionally, they could reach the totally daily intake (TDI) established even with very low levels of Besides, they could reach the totally daily intake (TDI) established even with very low levels of contamination in baby foods [41,42]. This fact must be taken into account especially in legislated mycotoxins because lower limits than those for adults have been set for baby food. Furthermore, another problem to deal with is that related to durum wheat pasta with small size, not specifically defined as baby food, but usually consumed by children. The most detected mycotoxin in baby food was DON [41,43]. Regarding emerging mycotoxins, in our study the only EN detected in baby food samples was ENA_1_ with an average amount of 1.54 µg/kg, contrary to durum wheat pasta where all ENs were detected and ENB showed higher contents (Table 3). In the study conducted by Juan et al. [41], ENB was also the EN with higher incidence in baby food (70%) with a maximum value of 1100.00 µg/kg. Serrano et al. [38] analyzed ENs and BEA in 45 cereal-based baby food and ENA, ENA_1_, ENB, and ENB_1_ were detected in 2.2%, 13.3%, 2.2%, and 40% samples at levels below 149.6, 101.7, 39.4, and 35.8 mg/kg, respectively.

According to available data, the co-occurrence of different mycotoxins in one sample at the same time has been widely described. Special attention should be paid to mycotoxin co-occurrence as different mixtures of TRC have been described to cause additive, antagonistic, or synergistic effects [24,44].

## 4. Conclusions

A multi-mycotoxin method has been applied in routine for mycotoxin screening and quantitation in pasta samples. In summary, by combining a validated QuEChERS extraction procedure with an UHPLC/ESI Q-orbitrap, an accurate and highly sensitive method has been developed to propose a useful approach to multi-residual analysis of mycotoxins in pasta samples.

## 5. Materials and Methods 

### 5.1. Chemical and Reagents

Mycotoxin standards, OTA, AFB_1_, NIV, NEO, DON, FUS-X, 15-AcDON, 3-AcDON, NEO, HT-2, T2, ZON, BEA, ENs (ENA, ENA_1_, and ENB), FB_1_, and FB_2_ were purchased from Sigma Aldrich (Milan, Italy). 

Acetonitrile (MeCN), methanol (MeOH), and water for LC mobile phase and organic solvents were HPLC grade from Merck (Darmstadt, Germany), while formic acid and ammonium formate were obtained from Fluka (Milan, Italy). Sodium chloride and magnesium sulphate were obtained from Sigma Aldrich (Milan, Italy). 

Syringe filters with polytetrafluoroethylene membrane (PTFE, 15 mm, diameter 0.2 mm) were provided by Phenomenex (Castel Maggiore, Italy). Corning PQ centrifuge polypropylene tubes of 50 and 15 mL (Corning Cable Systems SRL, Turin, Italy) were used.

### 5.2. Analytical Standards

The individual stock solutions of mycotoxins (AFB_1_, OTA, NEO, HT-2, T-2, DON, 3-AcDON, 15-AcDON, NIV, ZON, BEA, FUS-X, and T-2, ENs (ENA, ENA_1_, ENB) were prepared by diluting 1 mg of each mycotoxin in 1 mL of MeCN. However, for FB_1_ and FB_2_ the standards were prepared in MeCN/H_2_O 50:50 *v*/*v* solution at 1000 mg/L. On the other hand, for preparing intermediate solutions (working standard solutions) the individual stock standards were diluted in the same mixture solvent at different concentration levels. All these solutions were kept in safe conditionsat −20 °C.

### 5.3. Samples

Occurrence of mycotoxins were analyzed in a total of 58 samples of different conventional pasta products collected from several local markets of Campania region (Italy) and analyzed in order to investigate the presence of 17 mycotoxins. In accordance to the Commission Regulation EC/401/2006 [45], before the analysis performance, all samples were milled with a knife mill (Grindomix GM 200, Retsch, Haan, Germany) and then, samples were grounded employing a high speed food blender (Ika, mod. A11 basic, Germany) in order to obtain a fine ground. Ground samples were mixed by hand and a 100 g portion was removed by manual scooping. The subsamples were stored in a dark and dry place at 4 °C until analysis. Finally, portions of 4 g were placed into a 50 mL PTFE centrifugal tube for extraction purposes.

### 5.4. Extraction Procedure

QuEChERS extraction has been widely used by different authors in cereal based foodstuffs, showing good results. The procedure followed in the present survey was based in that reported by Zachariasova et al. [19]. Briefly: 4 g of homogenous representative sample were weighted into the PTFE cuvette and 7.5 mL of 0.1% (*v*/*v*) formic acid and 10mL of MeCN were added. The suspension was shaken vigorously for 3min. After addition of 1 g of NaCl and 4 g of MgSO_4_, the mixture was shaken again. To separate aqueous and organic phase, the sample was centrifuged (5 min, 5000 rpm (1960 g)). The 0.5 mL aliquot of upper organic phase was diluted with deionized water in 1:1 (*v*/*v*) ratio. The sample solution was filtered through the 0.2 µm filter prior to instrumental analysis.

### 5.5. Instruments and Analytical conditions

#### 5.5.1. UHPLC Chromatographic Analysis

Qualitative and quantitative profile of mycotoxins has been obtained using ultra-high-performance liquid chromatography (UHPLC, Thermo Fisher Scientific, Waltham, MA, USA) equipped with a degassing system, a Dionex Ultimate 3000 a Quaternary UHPLC pump working at 1250 bar, an auto sampler device and a thermostated (T = 50 °C) Accucore aQ C18 column (100 × 2.1 mm 2.6 µm particle size), (Thermo Fisher Scientific, Bellefonte, PA, USA). Injection volume was 5 µL. Eluent phase was formed as follows: phase A (H_2_O in 0.1% formic acid and 5mM ammonium formate), phase B (methanol in 0.1% formic acid and 5mM ammonium formate). Analytes have been eluted using a 0.5 mL/min flow rate with the following programmed gradient: 0 min—60% of phase B, 9 min—100% of phase B, 12 min—100% of phase B, 12.1 min—60% of phase B, 15 min—60% of phase B.

#### 5.5.2. High-Resolution Mass Spectrometry Analysis: Q Exactive Orbitrap Mass Spectrometry Analysis

For the mass spectrometry analysis a Q Exactive Orbitrap LC-MS/MS (Thermo Fisher Scientific, Waltham, MA, USA) has been used. An electrospray ionization ESI source (HESI II, Thermo Fischer Scientific, Waltham, MA, USA) operating in negative (ESI^−^) and in positive (ESI^+^) ion modes for all the analyzed compounds. Ion source parameters in both ESI− and ESI+ mode were: spray voltage 3.50 kV, sheath gas (N_2_> 95%) 30, auxiliary gas (N_2_> 95%) 10, capillary temperature 320°C, S-lens RF level 50, auxiliary gas heater temperature 300°C. 

Value for automatic gain control (AGC) target was set at 1 × 10^6^, with a resolution of 70,000 FWHM (full width at half maximum), isolation window to 5.0 *m*/*z*, and a scan rate in the range between 90 and 1000 *m*/*z* in full MS/scan mode.

The accuracy and calibration of the Q Exactive Orbitrap LC-MS/MS was checked weekly using a reference standard mixture obtained from Thermo Fisher Scientific. Data processing has been performed using the Xcalibur software, v. 3.0.63 (Xcalibur, Thermo Fisher Scientific, Waltham, MA, USA).

### 5.6. Data Analysis

Mycotoxins were identified by their retention time from the extracted ion chromatogram (XIC) of target ion *m*/*z* for each mycotoxin and the exact mass set to five decimal places. The mass accuracy (δM) for a measured ion has been calculated by dividing the difference between the theoretical and measured *m*/*z* by the theoretical m/z and expressed as part-per-million (ppm):

$$\delta M\;\left(\mathrm{ppm}\right)\;=1\times 10^{6}\frac{\left(m/z_{\mathrm{measured}}-m/z_{\mathrm{theoretical}}\right)}{m/z_{\mathrm{theoretical}}}$$

### 5.7. Analytical Parameters

#### 5.7.1. Recovery Studies

The accuracy of the extraction method was evaluated with the recovery test as the ratio of the mean observed concentration and the known spiked concentration in both durum wheat pasta and baby food, and was expressed as [(mean observed concentration)/(added concentration)] × 100 and the results were corrected by the diluting factor. Method recovery from two different homogenized samples was performed at three spiking levels (125 µg/kg, 62.5 µg/kg and 12.5 µg/kg), except for FB_2_, which was calculated at two spiking levels (125 µg/kg, and 62.5 µg/kg). To achieve these fortification levels, different aliquots from the 10 mg/kg standards were added to 4 g of grounded samples. After the standard addition, samples were placed overnight to allow solvent evaporation and to establish equilibration between the analytes and the matrix, and then were extracted as described in Section 5.4. Extraction Procedure.

#### 5.7.2. Matrix Effect Studies

To assess the possible matrix effect on the chromatographic response, the matrix effect (signal enhancement or suppression) was investigated by calculating the ratio percentage between the slopes of the matrix-matched calibration curve and the curve in solvent as follows:

$$\%\mathrm{ME}=\;\left(1-\frac{Sm}{Ss}\right)\times 100$$

where *Sm* is the slope of calibration curve in matrix-matched calibration solution and *Ss* is the slope of calibration curve with solvent. Solvent employed to calculate ME was MeCN, except for FUMs, which solvent employed was MeCN/H2O 50:50 *v*/*v*. Negative results were obtained when signal suppression occurs, while positive results corresponded to signal enhancement due to matrix effects.

#### 5.7.3. LOD, LOQ, and Linearity

Sensitivity was evaluated by limit of detection (LOD) and limit of quantitation (LOQ) values. The LODs and LOQs were determined by analyzing spiked samples and expressed as the ratio between standard deviation of three replicates measurements at low concentrations and the slope of the linear calibration curve generated at those low levels. LOQ was calculated as three times the LOD. Thus, LOD and LOQ were calculated according to the following formula:

$$\mathrm{LOD}\;\left(\frac{µg}{\mathrm{kg}}\right)=\frac{\mathrm{STD}\;\mathrm{areas}\;\mathrm{lowest}\;\mathrm{detection}\;\mathrm{level}}{\mathrm{slope}}\times 3\;\mathrm{LOQ}\;\left(\frac{µg}{\mathrm{kg}}\right)=\;\mathrm{LOD}\times 3$$

Linearity was evaluated for each mycotoxin using the calibration curve of each standard at different concentration levels, which ranged from 0.25 µg/kg to 1000 µg/kg for all analyzed mycotoxins.

## Figures and Tables

**Table 1:** Ultra high performance liquid chromatography (UHPLC)/Q-Orbitrap parameters for the 17 mycotoxins analyzed.

Mycotoxin	Retention Time (min)	Elemental Composition	Adduct Ion	Theoretical Mass (*m*/*z*)	Measured (*m*/*z*)	Accuracy (Δppm)
Neosolaniol	0.49	C19H26O8	[M + NH_4_]^+^	400.19659	400.19638	−0.52
Fusarenon-X	0.47	C17H22O8	[M + H]^+^	355.13874	355.13846	−0.79
Nivalenol	0.52	C15H20O7	[M + FAc]^−^	357.11911	357.11771	−3.92
Deoxynivalenol	0.56	C15H20O6	[M + FAc]^−^	341.12419	341.12378	−1.20
Sum of 3- and 15-AcDon	0.65	C17H22O7	[M + Na]^+^	361.12577	361.12589	0.33
HT-2 toxin	0.93	C22H32O8	[M + Na]^+^	447.19894	447.19916	0.49
T-2 toxin	1.22	C24H34O9	[M + Na]^+^	489.20950	489.20978	0.57
Zearalenone	1.55	C18H22O5	[M + H]^+^	319.15400	319.15375	−0.78
Aflatoxin B_1_	0.71	C17H12O6	[M + H]^+^	313.07066	313.07014	−1.66
Fumonisin B_1_	1.00	C34H59NO15	[M + H]^+^	722.39574	722.39396	−2.46
Fumonisin B_2_	2.47	C34H59NO14	[M + H]^+^	706.40083	706.40215	1.87
Enniatin A	7.71	C36H63N3O9	[M + NH_4_]^+^	699.49026	699.49048	0.31
Enniatin A_1_	7.32	C35H61N3O9	[M + NH_4_]^+^	685.47461	685.47449	−0.18
Enniatin B	6.37	C33H57N3O9	[M + H]^+^	640.41676	640.41930	3.97
Beauvericin	7.18	C45H57N3O9	[M + Na]^+^	806.39870	806.39697	−2.14
Ochratoxin A	1.53	C20H18NO6Cl	[M + H]^+^	404.08954	404.08925	−0.72

**Table 2:** Validation results in terms of recovery, matrix effect (ME, expressed in %), limits of detection and quantitation (LOD and LOQ, respectively), linearity (expressed as “r^2^”) and calibration curve for each mycotoxin.

Mycotoxin	Recovery(%)	ME (%)	LOD (µg/kg)	LOQ (µg/kg)	*r*^2^	Calibration Curve
Neosolaniol	68	36	0.03	0.11	0.9988	*Y* = −1.09137 × 10^6^ + 644247*X*
Fusarenon-X	99	55	2.80	8.42	0.9982	*Y* = −35936.3 + 14855.6*X*
Nivalenol	74	35	0.29	0.87	0.9996	*Y* = −75591.9 + 127604*X*
Deoxynivalenol	89	57	0.03	0.09	0.9989	*Y* = −199036 + 76100.7*X*
Sum of 3- and 15-AcDon	135	102	0.45	1.37	0.9956	*Y* = 2.29667 × 10^6^ + 629614*X*
HT-2 toxin	118	68	0.15	0.45	0.9993	*Y* = −569704 + 407261*X*
T-2 toxin	149	57	0.23	0.70	0.9997	*Y* = −1.7152 × 10^6^ + 1.58156 × 10^6^*X*
Zearalenone	148	86	0.21	0.65	0.9996	*Y* = −575778 + 340107*X*
Aflatoxin B_1_	128	64	0.03	0.11	0.9998	*Y* = 668377 + 6.27115 × 10^6^*X*
Fumonisin B_1_	105	72	1.65	4.95	0.9939	*Y* = −298323 + 62417*X*
Fumonisin B_2_	132	85	8.21	24.6	0.9864	*Y* = −655884 + 70612.3*X*
Enniatin A	165	105	0.39	1.19	0.9999	*Y* = −186911 + 1.92893 × 10^6^*X*
Enniatin A_1_	148	90	0.28	0.85	0.9999	*Y* = −486269 + 1.68215 × 10^6^*X*
Enniatin B	123	89	1.08	3.26	0.9971	*Y* = −255826 + 47880*X*
Beauvericin	210	17	0.89	2.68	0.9992	*Y* = −483543 + 157248*X*
Ochratoxin A	139	91	0.07	0.22	0.9986	*Y* = −717566 + 224556*X*

**Table 3:** Incidence and mycotoxin contents in samples analyzed, and MLs established for cereal foodstuffs.

Mycotoxin	Incidence(%)	Range(Mean) (µg/kg)	IARC Classification	MLs Established byCommission Regulation (EC) No. 1126/2007 for Cereals (µg/kg)
Neosolaniol	ND	ND	NC	No limits established
Fusarenon-X	ND	ND	3	No limits established
Nivalenol	93.33	11.54–16.90 (13.87)	3	No limits established
Deoxynivalenol	100.00	20.89–247.27 (96.93)	3	<750
Sum of 3- and 15-AcDon	86.67	1.46–4.18 (2.47)	NC	No limits established
HT-2 toxin	90.00	10.92–14.60 (12.46)	NC	No limits established
T-2 toxin	76.67	36.45–38.02 (36.95)	NC	No limits established
Zearalenone	93.30	16.84–19.94 (17.54)	3	20 *–75
Aflatoxin B_1_	ND	ND	1	0.1 *–2
Fumonisin B_1_	ND	ND	2B	200–1000 **
Fumonisin B_2_	ND	ND	2B	
Enniatin A	33.30	4.25–5.09 (4.46)	NC	No limits established
Enniatin A_1_	93.30	4.47–18.83 (9.83)	NC	No limits established
Enniatin B	90.00	91.15–710.90 (326.17)	NC	No limits established
Beauvericin	10.00	53.66–73.67 (63.66)	NC	No limits established
Ochratoxin A	ND	ND	2B	0.5 *–3

*: values reported for infants; **: limits referred to maize products; ND: not detected; NC: not classified.
